# DNA Glycosylases Involved in Base Excision Repair May Be Associated with Cancer Risk in *BRCA1* and *BRCA2* Mutation Carriers

**DOI:** 10.1371/journal.pgen.1004256

**Published:** 2014-04-03

**Authors:** Ana Osorio, Roger L. Milne, Karoline Kuchenbaecker, Tereza Vaclová, Guillermo Pita, Rosario Alonso, Paolo Peterlongo, Ignacio Blanco, Miguel de la Hoya, Mercedes Duran, Orland Díez, Teresa Ramón y Cajal, Irene Konstantopoulou, Cristina Martínez-Bouzas, Raquel Andrés Conejero, Penny Soucy, Lesley McGuffog, Daniel Barrowdale, Andrew Lee, SWE-BRCA, Brita Arver, Johanna Rantala, Niklas Loman, Hans Ehrencrona, Olufunmilayo I. Olopade, Mary S. Beattie, Susan M. Domchek, Katherine Nathanson, Timothy R. Rebbeck, Banu K. Arun, Beth Y. Karlan, Christine Walsh, Jenny Lester, Esther M. John, Alice S. Whittemore, Mary B. Daly, Melissa Southey, John Hopper, Mary B. Terry, Saundra S. Buys, Ramunas Janavicius, Cecilia M. Dorfling, Elizabeth J. van Rensburg, Linda Steele, Susan L. Neuhausen, Yuan Chun Ding, Thomas v. O. Hansen, Lars Jønson, Bent Ejlertsen, Anne-Marie Gerdes, Mar Infante, Belén Herráez, Leticia Thais Moreno, Jeffrey N. Weitzel, Josef Herzog, Kisa Weeman, Siranoush Manoukian, Bernard Peissel, Daniela Zaffaroni, Giulietta Scuvera, Bernardo Bonanni, Frederique Mariette, Sara Volorio, Alessandra Viel, Liliana Varesco, Laura Papi, Laura Ottini, Maria Grazia Tibiletti, Paolo Radice, Drakoulis Yannoukakos, Judy Garber, Steve Ellis, Debra Frost, Radka Platte, Elena Fineberg, Gareth Evans, Fiona Lalloo, Louise Izatt, Ros Eeles, Julian Adlard, Rosemarie Davidson, Trevor Cole, Diana Eccles, Jackie Cook, Shirley Hodgson, Carole Brewer, Marc Tischkowitz, Fiona Douglas, Mary Porteous, Lucy Side, Lisa Walker, Patrick Morrison, Alan Donaldson, John Kennedy, Claire Foo, Andrew K. Godwin, Rita Katharina Schmutzler, Barbara Wappenschmidt, Kerstin Rhiem, Christoph Engel, Alfons Meindl, Nina Ditsch, Norbert Arnold, Hans Jörg Plendl, Dieter Niederacher, Christian Sutter, Shan Wang-Gohrke, Doris Steinemann, Sabine Preisler-Adams, Karin Kast, Raymonda Varon-Mateeva, Andrea Gehrig, Dominique Stoppa-Lyonnet, Olga M. Sinilnikova, Sylvie Mazoyer, Francesca Damiola, Bruce Poppe, Kathleen Claes, Marion Piedmonte, Kathy Tucker, Floor Backes, Gustavo Rodríguez, Wendy Brewster, Katie Wakeley, Thomas Rutherford, Trinidad Caldés, Heli Nevanlinna, Kristiina Aittomäki, Matti A. Rookus, Theo A. M. van Os, Lizet van der Kolk, J. L. de Lange, Hanne E. J. Meijers-Heijboer, A. H. van der Hout, Christi J. van Asperen, Encarna B. Gómez Garcia, Nicoline Hoogerbrugge, J. Margriet Collée, Carolien H. M. van Deurzen, Rob B. van der Luijt, Peter Devilee, HEBON, Edith Olah, Conxi Lázaro, Alex Teulé, Mireia Menéndez, Anna Jakubowska, Cezary Cybulski, Jacek Gronwald, Jan Lubinski, Katarzyna Durda, Katarzyna Jaworska-Bieniek, Oskar Th. Johannsson, Christine Maugard, Marco Montagna, Silvia Tognazzo, Manuel R. Teixeira, Sue Healey, KConFab Investigators, Curtis Olswold, Lucia Guidugli, Noralane Lindor, Susan Slager, Csilla I. Szabo, Joseph Vijai, Mark Robson, Noah Kauff, Liying Zhang, Rohini Rau-Murthy, Anneliese Fink-Retter, Christian F. Singer, Christine Rappaport, Daphne Geschwantler Kaulich, Georg Pfeiler, Muy-Kheng Tea, Andreas Berger, Catherine M. Phelan, Mark H. Greene, Phuong L. Mai, Flavio Lejbkowicz, Irene Andrulis, Anna Marie Mulligan, Gord Glendon, Amanda Ewart Toland, Anders Bojesen, Inge Sokilde Pedersen, Lone Sunde, Mads Thomassen, Torben A. Kruse, Uffe Birk Jensen, Eitan Friedman, Yael Laitman, Shani Paluch Shimon, Jacques Simard, Douglas F. Easton, Kenneth Offit, Fergus J. Couch, Georgia Chenevix-Trench, Antonis C. Antoniou, Javier Benitez

**Affiliations:** 1 Human Genetics Group, Spanish National Cancer Centre (CNIO), Madrid, Spain; 2 Biomedical Network on Rare Diseases (CIBERER), Madrid, Spain; 3 Cancer Epidemiology Centre, Cancer Council Victoria, Melbourne, Australia; 4 Centre for Cancer Genetic Epidemiology, Department of Public Health and Primary Care, University of Cambridge, Cambridge, United Kingdom; 5 Genotyping Unit (CeGen), Spanish National Cancer Centre (CNIO), Madrid, Spain; 6 IFOM, Fondazione Istituto FIRC di Oncologia Molecolare, Milan, Italy; 7 Genetic Counseling Unit, Hereditary Cancer Program, IDIBELL-Catalan Institute of Oncology, Barcelona, Spain; 8 Molecular Oncology Laboratory, Hospital Clinico San Carlos, IdISSC, Madrid, Spain; 9 Institute of Biology and Molecular Genetics, Universidad de Valladolid (IBGM-UVA), Valladolid, Spain; 10 Oncogenetics Laboratory, University Hospital Vall d'Hebron, Vall d'Hebron Institute of Oncology (VHIO), Vall d'Hebron Institut de Recerca (VHIR), and Universitat Autonoma de Barcelona, Barcelona, Spain; 11 Oncology Service, Hospital de la Santa Creu i Sant Pau, Barcelona, Spain; 12 Molecular Diagnostics Laboratory IRRP, National Centre for Scientific Research Demokritos Aghia Paraskevi Attikis, Athens, Greece; 13 Molecular Genetics Laboratory (Department of Biochemistry), Cruces Hospital Barakaldo, Bizkaia, Spain; 14 Medical Oncology Service, Hospital Clínico Lozano Blesa, San Juan Bosco, Zaragoza, Spain; 15 Cancer Genomics Laboratory, Centre Hospitalier Universitaire de Québec and Laval University, Quebec City, Canada; 16 Department of Oncology, Lund University, Lund, Sweden; 17 Department of Oncology, Karolinska University Hospital, Stockholm, Sweden; 18 Department of Clinical Genetics, Karolinska University Hospital, Stockholm, Sweden; 19 Department of Oncology, Lund University Hospital, Lund, Sweden; 20 Department of Clinical Genetics, Lund University Hospital, Lund, Sweden; 21 Center for Clinical Cancer Genetics and Global Health, University of Chicago Medical Center, Chicago, Illinois, United States of America; 22 Departments of Medicine, Epidemiology, and Biostatistics, University of California, San Francisco, San Francisco, California, United States of America; 23 Abramson Cancer Center and Department of Medicine, The University of Pennsylvania School of Medicine, Philadelphia, Pennsylvania, United States of America; 24 Abramson Cancer Center and Center for Clinical Epidemiology and Biostatistics, The University of Pennsylvania Perelman School of Medicine, Philadelphia, Pennsylvania, United States of America; 25 University of Texas MD Anderson Cancer Center, Houston, Texas, United States of America; 26 Women's Cancer Program at the Samuel Oschin Comprehensive Cancer Institute, Cedars-Sinai Medical Center, Los Angeles, California, United States of America; 27 Department of Epidemiology, Cancer Prevention Institute of California, Fremont, California, United States of America; 28 Department of Health Research & Policy, Stanford University School of Medicine, Stanford, California, United States of America; 29 Fox Chase Cancer Center, Philadelphia, Pennsylvania, United States of America; 30 Genetic Epidemiology Laboratory, Department of Pathology, University of Melbourne, Parkville, Australia; 31 Centre for Molecular, Environmental, Genetic and Analytic Epidemiology, University of Melbourne, Melbourne, Victoria, Australia; 32 Department of Epidemiology, Columbia University, New York, New York, United States of America; 33 Department of Oncological Sciences, Huntsman Cancer Institute, University of Utah School of Medicine, Salt Lake City, Utah, United States of America; 34 Vilnius University Hospital Santariskiu Clinics, Hematology, oncology and transfusion medicine center, Department of Molecular and Regenerative Medicine, Vilnius, Lithuania; 35 Department of Genetics, University of Pretoria, Pretoria, South Africa; 36 Department of Population Sciences, Beckman Research Institute of City of Hope, Duarte, California, United States of America; 37 Center for Genomic Medicine, Rigshospitalet, University of Copenhagen, Copenhagen, Denmark; 38 Department of Oncology, Rigshospitalet, University of Copenhagen, Copenhagen, Denmark; 39 Department of Clinical Genetics, Rigshospitalet, University of Copenhagen, Copenhagen, Denmark; 40 Clinical Cancer Genetics, City of Hope, Duarte, California, United States of America; 41 Unit of Medical Genetics, Department of Preventive and Predictive Medicine, Fondazione IRCCS Istituto Nazionale Tumori (INT), Milan, Italy; 42 Division of Cancer Prevention and Genetics, Istituto Europeo di Oncologia, Milan, Italy; 43 IFOM, Fondazione Istituto FIRC di Oncologia Molecolare and Cogentech Cancer Genetic Test Laboratory, Milan, Italy; 44 Division of Experimental Oncology 1, Centro di Riferimento Oncologico, IRCCS, Aviano, Italy; 45 Unit of Hereditary Cancer, Department of Epidemiology, Prevention and Special Functions, IRCCS AOU San Martino - IST Istituto Nazionale per la Ricerca sul Cancro, Genoa, Italy; 46 Unit of Medical Genetics, Department of Biomedical, Experimental and Clinical Sciences, University of Florence, Florence, Italy; 47 Department of Molecular Medicine, “Sapienza” University, Rome, Italy; 48 UO Anatomia Patologica, Ospedale di Circolo-Università dell'Insubria, Varese, Italy; 49 Unit of Molecular bases of genetic risk and genetic testing, Department of Preventive and Predictive Medicine, Fondazione IRCCS Istituto Nazionale Tumori (INT), Milan, Italy; 50 Dana-Farber Cancer Institute, Boston, Massachusetts, United States of America; 51 Genetic Medicine, Manchester Academic Health Sciences Centre, Central Manchester University Hospitals NHS Foundation Trust, Manchester, United Kingdom; 52 South East Thames Regional Genetics Service, Guy's Hospital London, United Kingdom; 53 Oncogenetics Team, The Institute of Cancer Research and Royal Marsden NHS Foundation Trust, London, United Kingdom; 54 Yorkshire Regional Genetics Service, Leeds, United Kingdom; 55 Ferguson-Smith Centre for Clinical Genetics, Yorkhill Hospitals, Glasgow, United Kingdom; 56 West Midlands Regional Genetics Service, Birmingham Women's Hospital Healthcare NHS Trust, Edgbaston, Birmingham, United Kingdom; 57 Wessex Clinical Genetics Service, Princess Anne Hospital, Southampton, United Kingdom; 58 Sheffield Clinical Genetics Service, Sheffield Children's Hospital, Sheffield, United Kingdom; 59 Clinical Genetics Department, St Georges Hospital, University of London, London, United Kingdom; 60 Department of Clinical Genetics, Royal Devon & Exeter Hospital, Exeter, United Kingdom; 61 Department of Clinical Genetics, East Anglian Regional Genetics Service, Addenbrookes Hospital, Cambridge, United Kingdom; 62 Institute of Human Genetics, Centre for Life, Newcastle Upon Tyne Hospitals NHS Trust, Newcastle upon Tyne, United Kingdom; 63 South East of Scotland Regional Genetics Service, Western General Hospital, Edinburgh, United Kingdom; 64 North East Thames Regional Genetics Service, Great Ormond Street Hospital for Children NHS Trust, London, United Kingdom; 65 Oxford Regional Genetics Service, Churchill Hospital, Oxford, United Kingdom; 66 Northern Ireland Regional Genetics Centre, Belfast City Hospital, Belfast, United Kingdom; 67 South West Regional Genetics Service, Bristol, United Kingdom; 68 Academic Unit of Clinical and Molecular Oncology, Trinity College Dublin and St James's Hospital, Dublin, Eire; 69 Cheshire & Merseyside Clinical Genetics Service, Liverpool Women's NHS Foundation Trust, Liverpool, United Kingdom; 70 Department of Pathology and Laboratory Medicine, University of Kansas Medical Center, Kansas City, Kansas, United States of America; 71 Centre of Familial Breast and Ovarian Cancer and Centre for Integrated Oncology (CIO), University Hospital of Cologne, Cologne, Germany; 72 Institute for Medical Informatics, Statistics and Epidemiology, University of Leipzig, Leipzig, Germany; 73 Department of Gynaecology and Obstetrics, Division of Tumor Genetics, Klinikum rechts der Isar, Technical University Munich, Munich, Germany; 74 Department of Gynecology and Obstetrics, University Medical Center Schleswig-Holstein, Campus Kiel, Kiel, Germany; 75 Institute of Human Genetics, University Medical Center Schleswig-Holstein, Campus Kiel, Kiel, Germany; 76 Department of Gynaecology and Obstetrics, University Hospital Düsseldorf, Heinrich-Heine University Düsseldorf, Düsseldorf, Germany; 77 Institute of Human Genetics, Department of Human Genetics, University Hospital Heidelberg, Heidelberg, Germany; 78 Department of Gynaecology and Obstetrics, University Hospital Ulm, Ulm, Germany; 79 Institute of Cell and Molecular Pathology, Hannover Medical School, Hannover, Germany; 80 Institute of Human Genetics, University of Münster, Münster, Germany; 81 Department of Gynaecology and Obstetrics, University Hospital Carl Gustav Carus, Technical University Dresden, Dresden, Germany; 82 Institute of Human Genetics, Campus Virchov Klinikum, Charite Berlin, Berlin, Germany; 83 Centre of Familial Breast and Ovarian Cancer, Department of Medical Genetics, Institute of Human Genetics, University Würzburg, Würzburg, Germany; 84 Institut Curie, Department of Tumour Biology, Paris, France; 85 Institut Curie, INSERM U830, Paris, France; 86 Université Paris Descartes, Sorbonne Paris Cité, Paris, France; 87 Unité Mixte de Génétique Constitutionnelle des Cancers Fréquents, Hospices Civils de Lyon – Centre Léon Bérard, Lyon, France; 88 INSERM U1052, CNRS UMR5286, Université Lyon 1, Centre de Recherche en Cancérologie de Lyon, Lyon, France; 89 Center for Medical Genetics, Ghent University, Ghent, Belgium; 90 Gynecologic Oncology Group Statistical and Data Center, Roswell Park Cancer Institute, Buffalo, New York, United States of America; 91 Prince of Wales Hospital. Sydney, Australia; 92 Ohio State University, Columbus Cancer Council, Columbus, Ohio, United States of America; 93 Division of Gynecologic Oncology, NorthShore University HealthSystem, Evanston, Illinois, United States of America; 94 Division of Gynecologic Oncology, NorthShore University HealthSystem, Chicago, Illinois, United States of America; 95 For Tufts Medical Center, Boston, Massachusetts, United States of America; 96 Yale University School of Medicine, New Haven, Connecticut, United States of America; 97 Department of Obstetrics and Gynecology, University of Helsinki and Helsinki University Central Hospital, Helsinki, Finland; 98 Department of Epidemiology, Netherlands Cancer Institute, Amsterdam, The Netherlands; 99 Department of Clinical Genetics, Academic Medical Center, Amsterdam, The Netherlands; 100 Family Cancer Clinic, Netherlands Cancer Institute, Amsterdam, The Netherlands; 101 Department of Epidemiology, Netherlands Cancer Institute, Amsterdam, The Netherlands; 102 Department of Clinical Genetics, VU University Medical Centre, Amsterdam, The Netherlands; 103 University of Groningen, University Medical Center Groningen, Department of Genetics, Groningen, The Netherlands; 104 Department of Clinical Genetics, Leiden University Medical Center Leiden, Leiden, The Netherlands; 105 Department of Clinical Genetics and GROW, School for Oncology and Developmental Biology, MUMC, Maastricht, The Netherlands; 106 Department of Human Genetics, Radboud University Nijmegen Medical Centre, Nijmegen, The Netherlands; 107 Department of Clinical Genetics, Family Cancer Clinic, Erasmus University Medical Center, Rotterdam, The Netherlands; 108 Department of Pathology, Family Cancer Clinic, Erasmus University Medical Center, Rotterdam, The Netherlands; 109 Department of Medical Genetics, University Medical Center Utrecht, Utrecht, The Netherlands; 110 Department of Human Genetics & Department of Pathology, Leiden University Medical Center, Leiden, The Netherlands; 111 The Hereditary Breast and Ovarian Cancer Research Group, Netherlands Cancer Institute, Amsterdam, The Netherlands; 112 Department of Molecular Genetics, National Institute of Oncology, Budapest, Hungary; 113 Molecular Diagnostic Unit, Hereditary Cancer Program, IDIBELL-Catalan Institute of Oncology, Barcelona, Spain; 114 Department of Genetics and Pathology, Pomeranian Medical University, Szczecin, Poland; 115 Postgraduate School of Molecular Medicine, Warsaw Medical University, Warsaw, Poland; 116 Department of Oncology, Landspitali University Hospital and BMC, Faculty of Medicine, University of Iceland, Reykjavik Iceland; 117 Laboratoire de Diagnostic Génétique et Service d'Onco-hématologie, Hopitaux Universitaire de Strasbourg, CHRU Nouvel Hôpital Civil, Strasbourg, France; 118 Immunology and Molecular Oncology Unit, Veneto Institute of Oncology IOV - IRCCS, Padua, Italy; 119 Department of Genetics, Portuguese Oncology Institute, Porto, and Biomedical Sciences Institute (ICBAS), Porto University, Porto, Portugal; 120 Department of Genetics and Computational Biology, Queensland Institute of Medical Research, Brisbane, Australia; 121 Kathleen Cuningham Consortium for Research into Familial Breast Cancer, Peter MacCallum Cancer Center, Melbourne, Australia; 122 Department of Health Sciences Research, Mayo Clinic, Rochester, Minnesota, United States of America; 123 Department of Laboratory Medicine and Pathology, Mayo Clinic, Rochester, Minnesota, United States of America; 124 Center for Individualized Medicine, Mayo Clinic, Scottsdale, Arizona, United States of America; 125 Department of Health Sciences Research, Mayo Clinic, Rochester, Minnesota, United States of America; 126 Center for Translational Cancer Research, Department of Biological Sciences, University of Delaware, Newark, Delaware, United States of America; 127 Clinical Genetics Service, Memorial Sloan-Kettering Cancer Center, New York, New York, United States of America; 128 Cancer Biology and Genetics Program, Memorial Sloan-Kettering Cancer Center, New York, New York, United States of America; 129 Diagnostic Molecular Genetics Laboratory, Memorial Sloan-Kettering Cancer Center, New York, New York, United States of America; 130 Department of OB/GYN and Comprehensive Cancer Center, Medical University of Vienna, Vienna, Austria; 131 Department of Cancer Epidemiology, Moffitt Cancer Center, Tampa, Florida, United States of America; 132 Clinical Genetics Branch, Division of Cancer Epidemiology and Genetics, National Cancer Institute, National Institutes of Health, Rockville, Maryland, United States of America; 133 Clalit National Israeli Cancer Control Center, Haifa, Israel; 134 Samuel Lunenfeld Research Institute, Mount Sinai Hospital, Toronto, Ontario, Canada, and Cancer Care Ontario, Departments of Molecular Genetics and Laboratory Medicine and Pathobiology, University of Toronto, Toronto, Ontario, Canada; 135 Department of Laboratory Medicine and Pathobiology, University of Toronto, Toronto, Ontario, Canada; Laboratory Medicine Program, University Health Network, Toronto, Ontario, Canada; 136 Ontario Cancer Genetics Network: Samuel Lunenfeld Research Institute, Mount Sinai Hospital, Toronto, Ontario, Canada; 137 Division of Human Cancer Genetics, Departments of Internal Medicine and Molecular Virology, Immunology and Medical Genetics, Comprehensive Cancer Center, The Ohio State University, Columbus, Ohio, United States of America; 138 Department of Clinical Genetics, Vejle Hospital, Vejle, Denmark; 139 Section of Molecular Diagnostics, Department of Clinical Biochemistry, Aalborg University Hospital, Aalborg, Denmark; 140 Department of Clinical Genetics, Aarhus University Hospital, Aarhus, Denmark; 141 Department of Clinical Genetics, Odense University Hospital, Odense, Denmark; 142 Sheba Medical Center, Tel Aviv, Israel; 143 Canada Research Chair in Oncogenetics, Cancer Genomics Laboratory, Centre Hospitalier Universitaire de Québec and Laval University, Quebec City, Canada; University of Washington, United States of America

## Abstract

Single Nucleotide Polymorphisms (SNPs) in genes involved in the DNA Base Excision Repair (BER) pathway could be associated with cancer risk in carriers of mutations in the high-penetrance susceptibility genes *BRCA1* and *BRCA2*, given the relation of synthetic lethality that exists between one of the components of the BER pathway, PARP1 (poly ADP ribose polymerase), and both BRCA1 and BRCA2. In the present study, we have performed a comprehensive analysis of 18 genes involved in BER using a tagging SNP approach in a large series of *BRCA1* and *BRCA2* mutation carriers. 144 SNPs were analyzed in a two stage study involving 23,463 carriers from the CIMBA consortium (the Consortium of Investigators of Modifiers of *BRCA1* and *BRCA2*). Eleven SNPs showed evidence of association with breast and/or ovarian cancer at p<0.05 in the combined analysis. Four of the five genes for which strongest evidence of association was observed were DNA glycosylases. The strongest evidence was for rs1466785 in the *NEIL2* (endonuclease VIII-like 2) gene (HR: 1.09, 95% CI (1.03–1.16), p = 2.7×10^−3^) for association with breast cancer risk in *BRCA2* mutation carriers, and rs2304277 in the *OGG1* (8-guanine DNA glycosylase) gene, with ovarian cancer risk in *BRCA1* mutation carriers (HR: 1.12 95%CI: 1.03–1.21, p = 4.8×10^−3^). DNA glycosylases involved in the first steps of the BER pathway may be associated with cancer risk in *BRCA1/2* mutation carriers and should be more comprehensively studied.

## Author Summary

Women harboring a germ-line mutation in the *BRCA1* or *BRCA2* genes have a high lifetime risk to develop breast and/or ovarian cancer. However, not all carriers develop cancer and high variability exists regarding age of onset of the disease and type of tumor. One of the causes of this variability lies in other genetic factors that modulate the phenotype, the so-called modifier genes. Identification of these genes might have important implications for risk assessment and decision making regarding prevention of the disease. Given that *BRCA1* and *BRCA2* participate in the repair of DNA double strand breaks, here we have investigated whether variations, Single Nucleotide Polymorphisms (SNPs), in genes participating in other DNA repair pathway may be associated with cancer risk in BRCA carriers. We have selected the Base Excision Repair pathway because BRCA defective cells are extremely sensitive to the inhibition of one of its components, PARP1. Thanks to a large international collaborative effort, we have been able to identify at least two SNPs that are associated with increased cancer risk in *BRCA1* and *BRCA2* mutation carriers respectively. These findings could have implications not only for risk assessment, but also for treatment of *BRCA1/2* mutation carriers with PARP inhibitors.

## Introduction

Carrying an inherited mutation in the *BRCA1* or *BRCA2* gene increases a woman's lifetime risk of developing breast, ovarian and other cancers. The estimated cumulative risk of developing breast cancer by the age of 70 in *BRCA1* and *BRCA2* mutation carriers varies between 43% to 88%; similarly, between 11% to 59% of mutation carriers will develop ovarian cancer by the age of 70 [1]–[3]. These considerable differences in disease manifestation suggest the existence of other genetic or environmental factors that modify the risk of cancer development. The Consortium of Investigators of Modifiers of *BRCA1* and *BRCA2* (CIMBA), was established in 2006 [4] and with more than 40,000 mutation carriers currently provides the largest sample size for reliable evaluation of even modest associations between single-nucleotide polymorphisms (SNPs) and cancer risk. CIMBA studies have so far demonstrated that more than 25 SNPs are associated with the risk of developing breast or ovarian cancer for *BRCA1* or *BRCA2* carriers. These were identified through genome-wide association studies (GWAS) of breast or ovarian cancer in the general population or through *BRCA1-* and *BRCA2-*specific GWAS [5]–[8]. Cells harboring mutations in *BRCA1* or *BRCA2* show impaired homologous recombination (HR) [9]–[11] and are thus critically dependent on other members of the DNA repair machinery such as poly ADP ribose polymerase (PARP1) involved in the Base Excision Repair (BER) pathway. The BER pathway is crucial for the replacement of aberrant bases generated by different causes [12]. A deficiency in BER can give rise to a further accumulation of double-strand DNA breaks which, in the presence of a defective *BRCA1* or *BRCA2* background, could persist and lead to cell cycle arrest or cell death; this makes BRCA-deficient cells extremely sensitive to PARP inhibitors, as previously demonstrated [13]. We hypothesize that SNPs in *PARP1* and other members of BER may be associated with cancer risk in *BRCA1* and *BRCA2* mutation carriers. SNPs in *XRCC1*, one of the main components of BER, have been recently evaluated within the CIMBA consortium [14], however a comprehensive study has not yet been performed of either *XRCC1* or the other genes participating in BER.

In the present study, we used a tagging SNP approach to evaluate whether the common genetic variation in the genes involved in the BER pathway could be associated with cancer risk in a large series of *BRCA1/*2 mutation carriers using a two-stage approach. The first stage involved an analysis of 144 tag SNPs in 1,787 Spanish and Italian *BRCA1/2* mutation carriers. In stage II, the 36 SNPs showing the strongest evidence of association in stage I, were evaluated in a further 23,463 CIMBA mutation carriers included in the Collaborative Oncological Gene-environment Study (COGS) and genotyped using the iCOGS custom genotyping array.

## Results

### Breast cancer association

**Figure 1:**
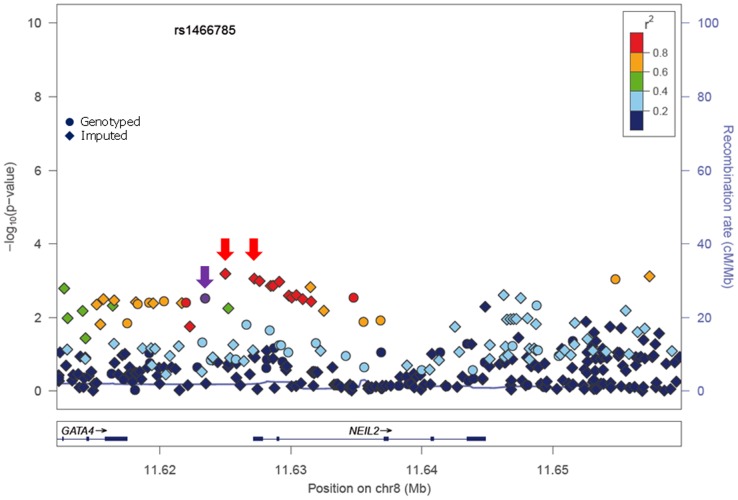
p-values of association (−log10 scale) with breast cancer risk in *BRCA2* carriers for genotyped and imputed SNPs in the *NEIL2* gene. SNP rs1466785 is indicated with a purple arrow and the best causal imputed SNPs, rs804276 and rs804271 are indicated with a red arrow. Colors represent the pariwise r^2^. Plot generated with LocusZoom [42] (http://csg.sph.umich.edu/locuszoom/).

In stage I, 144 selected Tag SNPs covering the 18 selected BER genes were genotyped in 968 *BRCA1* and 819 *BRCA2* mutation carriers from five CIMBA centres (Spanish National Cancer ResearchCentre (CNIO), Hospital Clínico San Carlos (HCSC), Catalan Institute of Oncology (ICO), Demokritos and Milan Breast Cancer Study Group (MBCSG). Of those, 50 were excluded because of low call-rates, minor allele frequency (MAF)<0.05, evidence of deviation from Hardy Weinberg Equilibrium (p-value<10^−3^) or monomorphism. Associations with breast cancer risk were assessed for 94 SNPs, as summarized in Table S1. The 36 SNPs that showed evidence of association at p≤0.05 were selected for analysis in stage II. Of the 36 SNPs successfully genotyped in the whole CIMBA series comprising 15,252 *BRCA1* and 8211 *BRCA2* mutation carriers, consistent evidence of association with breast cancer risk (p-trend<0.05) was observed for six SNPs (Table 1). The strongest evidence of association was observed for rs1466785 in the *NEIL2* gene (HR: 1.09, 95% CI (1.03–1.16), p = 2.7×10^−3^) for association with breast cancer risk in *BRCA2* mutation carriers. We had observed a consistent association in stage I in *BRCA2* mutation carriers (HR: 1.25, p = 0.06). The SNP was primarily associated with ER-negative breast cancer (HR: 1.20, 95%CI (1.06–1.37), p = 4×10^−3^), although the difference in HRs for ER-positive and ER-negative disease was not statistically significant. The evidence of association in Stage II was somewhat stronger when considering the genotype-specific models, with the dominant being the best fitting (HR: 1.20 95% CI: 1.09–1.37, p = 1×10^−4^). The associations remained significant and the estimated effect sizes remained consistent with the overall analysis when the data were reanalyzed excluding samples used in stage I of the study (data not shown). Imputation using the 1000 genomes data showed that there were several SNPs in strong linkage disequilibrium (LD) with rs1466785 showing more significant associations (p<10^−3^) (Figure 1).

**Table 1:** Associations with breast and ovarian cancer risk for SNPs observed at p-trend<0.05 in stage II of the experiment.

*BRCA1* carriers	SNP name	Gene	Unaffected (Number)	Affected (Number)	Unaffected (MAF)	Affected (MAF)	HR per allele^a^	HR heterozygote^b^	HR homozygote^b^	p-trend^c^	p-het^c^	p-hom^c^
**Breast cancer**	rs3847954^d^	*UNG*	7455	7797	0.18	0.19	1.05 (1.00–1.11)	1.09 (1.02–1.16)	0.99 (0.84–1.16)	0.04	0.011	0.713
**Ovarian cancer**	rs2072668	*OGG1*	12786	2461	0.22	0.23	1.09 (1.01–1.18)	1.16 (1.05–1.27)	1.03 (0.82–1.28)	0.016	3×10^−3^	0.77
	rs2269112	*OGG1*	12789	2461	0.17	0.18	1.11 (1.02–1.21)	1.11 (1.01–1.23)	1.21 (0.92–1.58)	0.013	0.014	0.268
	**rs2304277**	***OGG1***	12783	2462	0.2	0.21	**1.12 (1.03–1.21)**	**1.19 (1.08–1.3)**	**1.01 (0.79–1.30)**	**4.8×10^−3^**	**6×10^−4^**	**0.69**
	rs10161263	*SMUG1*	12790	2462	0.34	0.32	0.92 (0.86–0.99)	0.88 (0.80–0.97)	0.90 (0.78–1.04)	0.024	9×10^−3^	0.49

*BRCA2* carriers	SNP name	Gene	Unaffected (Number)	Affected (Number)	Unaffected (MAF)	Affected (MAF)	HR per allele	HR heterozygote	HR homozygote	p-trend	p-het	p-hom
**Breast cancer**	rs2072668^e^	*OGG1*	3879	4328	0.23	0.21	0.91 (0.85–0.98)	0.95 (0.87–1.04)	0.75 (0.62–0.91)	0.018	0.098	7×10^−3^
	rs2269112^f^	*OGG1*	3880	4329	0.17	0.16	0.91 (0.84–0.99)	0.93 (0.85–1.03)	0.76 (0.58–0.99)	0.035	0.083	0.054
	rs3136811^g^	*POLB*	3873	4321	0.06	0.07	1.12 (1.005–1.25)	1.17 (1.03–1.32)	0.86 (0.49–1.48)	0.032	0.019	0.715
	rs2304277^h^	*OGG1*	3880	4330	0.21	0.19	0.91 (0.84–0.97)	0.94 (0.85–1.03)	0.74 (0.60–0.91)	0.013	0.058	0.01
	**rs1466785** i	***NEIL2***	3879	4330	0.4	0.43	**1.09 (1.03–1.16)**	**1.20 (1.09–1.37)**	**1.16 (1.03–1.31)**	**2.7×10^−3^**	**1×10^−4^**	**0.455**
**Ovarian cancer**	**rs167715**	***TDG***	7577	631	0.12	0.09	**0.76 (0.62–0.94)**	**0.72 (0.58–0.90)**	**0.89 (0.41–1.89)**	**7.4×10^−3^**	**4.1×10^−3^**	**0.866**
	**rs3093926** j	***PARP2***	7580	631	0.07	0.05	**0.64 (0.49–0.84)**	**_**	**_**	**1.5×10^−3^**	**_**	**_**
	**rs4135087**	***TDG***	7580	631	0.09	0.11	**1.32 (1.09–1.59)**	**1.33 (1.07–1.65)**	**1.67 (0.84–3.28)**	**2.8×10^−3^**	**3.8×10^−3^**	**0.185**
	**rs34259**	***UNG***	7580	631	0.2	0.17	**0.80 (0.69–0.94)**	**0.84 (0.70–1.01)**	**0.51 (0.29–0.90)**	**7.6×10^−3^**	**0.025**	**0.028**

a Hazard Ratio per allele (1 df) estimated from the retrospective likelihood analysis.

b Hazard Ratio under the genotype specific models (2df) estimated from the retrospective likelihood analysis.

c p-values were based on the score test.

d HR per allele of 1.69 and p-trend of 1×10^−4^ for *BRCA2* mutation carriers in stage I of the study.

e HR per allele of 1.43 and p-trend of 0.01 for *BRCA1* mutation carriers in stage I of the study.

f HR per allele of 1.30 and p-trend of 0.03 for *BRCA1* mutation carriers in stage I of the study.

g HR per allele of 0.64 and p-trend of 0.057 for *BRCA2* mutation carriers in stage I of the study.

h HR per allele of 1.25 and p-trend of 0.04 for *BRCA1* mutation carriers in stage I of the study.

i HR per allele of 1.25 and p-trend of 0.058 for *BRCA2* mutation carriers in stage I of the study.

j rs3093926 did not yield results under the genotype specific model due to the low minor allele frequency.

Complete description of results from stage I are included in Supplementary Table S1.

Highlighted in bold are those SNPs showing strongest associations with breast or ovarian cancer risk (p<0.01).

### Ovarian cancer association

Due to lack of power we did not perform analysis of associations with ovarian cancer in stage I. However, we performed this analysis for the 36 SNPs tested in stage II. Although they had been selected based on their evidence of association with breast cancer risk, under the initial hypothesis they are also plausible modifiers of ovarian cancer risk for *BRCA1* and *BRCA2* mutation carriers. We found four SNPs associated with ovarian cancer risk with a p-trend<0.01 in *BRCA1* or *BRCA2* mutation carriers (Table 1). The strongest association was found for rs2304277 in *OGG1* in *BRCA1* mutation carriers (HR: 1.12, 95%CI: 1.03–1.21, p = 4.8×10^−3^). The association was somewhat stronger under the dominant model (HR: 1.19, 95%CI: 1.08–1.3, p = 6×10^−4^). Although three other SNPs were found to be associated with ovarian cancer risk in *BRCA2* mutation carriers (p-trend<10^−3^), these results were based on a relatively small number of ovarian cancer cases. Imputed data did not show any SNPs with substantially more significant associations with ovarian cancer risk except for rs3093926 in *PARP2*, associated with ovarian cancer risk in *BRCA2* mutation carriers for which there was a SNP, rs61995542, with a stronger association (HR: 0.67, p = 4.6×10^−4^) (Figure S1).

## Discussion

Based on the interaction of synthetic lethality that has been described between *PARP1* and both *BRCA1* and *BRCA2*, we hypothesize that this and other genes involved in the BER pathway could potentially be associated with cancer risk in *BRCA1/2* mutation carriers. Several studies have recently investigated the association of some of the BER genes with breast cancer, however, no definitive conclusions can be drawn, given that some publications suggest that SNPs in these genes can be associated with breast cancer risk with marginal p-values while others rule out a major role of these genes in the disease [15]–[21]. There is only one study from the CIMBA consortium which has evaluated the role of three of the most studied SNPs in the *XRCC1* gene, c.-77C>T (rs3213245) p.Arg280His (rs25489) and p.Gln399Arg (rs25487), ruling out associations of these variants with cancer risk in *BRCA1* and *BRCA2* mutation carriers [14]. However, a comprehensive analysis of neither *XRCC1* nor the other genes involved in the pathway in the context of BRCA mutation carriers has been performed. In the present study we have assessed the common genetic variation of 18 genes participating in BER by using a two stage strategy.

Eleven SNPs showed evidence of association with breast and/or ovarian cancer at p<0.05 in stage II of the experiment (Table 1). Of those, six showed a p-trend value<0.01 and were therefore considered the best candidates for further evaluation. Only one of those six, rs1466785 in the *NEIL2* gene (endonuclease VIII-like 2) showed an association with breast cancer risk while the other five, rs2304277 in *OGG1* (8-guanine DNA glycosylase), rs167715 and rs4135087 in *TDG* (thymine-DNA glycosylase), rs3093926 in *PARP2* (Poly(ADP-ribose) polymerase 2) and rs34259 in *UNG* (uracil-DNA glycosylase) were associated with ovarian cancer risk.

The minor allele of *NEIL2-*rs1466785 was associated with increased breast cancer risk in *BRCA2* mutation carriers; moreover, when considering the genotype-specific risks observed that the best fitting model was the dominant one. *NEIL2* is one of the oxidized base-specific DNA glycosylases that participate in the initial steps of BER and specifically removes oxidized bases from transcribing genes [22]. By imputing using the 1000 genome data we found six correlated SNPs in strong LD with rs1466785 (r^2^>0.8), located closer or inside the gene and showing slightly stronger and more significant associations with the disease and therefore being better candidate causal variants. From those, we considered rs804276 and rs804271 as the best candidates given that they showed the most significant associations (p = 6×10^−4^ and p = 8×10^−4^ respectively) and there were available epidemiological or functional data supporting their putative role in cancer. SNP rs804276 has been associated with disease recurrence in patients with bladder cancer treated with Bacillus Calmette-Guérin (BCG) (HR: 2.71, 95%CI (1.75–4.20), p = 9×10^−6^) [23]. SNP rs804271 is located in a positive regulatory region in the promoter of the gene, between two potential cis- binding sites for reactive oxygen species responsive transcription factors in which sequence variation has been proven to alter the transcriptional response to oxidative stress [24]. Moreover, this SNP has been proposed to partly explain the inter-individual variability observed in *NEIL2* expression levels in the general population and has been proposed as a potential risk modifier of disease susceptibility [25].

Several studies have been published showing associations between SNPs in *NEIL2* and lung or oropharyngeal cancer risk [26], [27] but to our knowledge, no association with breast cancer risk has been reported. We hypothesize that the potential association observed in the present study could be explained by the interaction between *NEIL2* and *BRCA2*, each of them causing a deficiency in the BER and HR DNA repair pathways, respectively. This would explain why the breast cancer risk modification due to rs1466785 would only be detected in the context of *BRCA2* mutation carriers and not in the general population.

The strongest evidence of association found in *BRCA1* carriers was between rs2304277 in the *OGG1* gene and ovarian cancer risk. The association was more significant when considering the dominant model. *OGG1* removes 8-oxodeoxyguanosine which is generated by oxidative stress and is highly mutagenic, and it has been suggested that SNPs in the gene could be associated with cancer risk [28]–[31]. This is an interesting result, given that to date only one SNP, rs4691139 in the 4q35.3 region, also identified through the iCOGS effort, has been found to modify ovarian cancer risk specifically in *BRCA1* carriers [32]. SNP rs2304277 is located in the 3′UTR (untranslated region) of the gene and is probably not the causal variant, however, in this case imputations through the 1000 Genome did not show better results for a more plausible causal SNP.

We have identified four SNPs associated with ovarian cancer risk in *BRCA2* mutation carriers, rs167715 and rs4135087 in the *TDG* gene, rs34259 in the *UNG* gene and rs3093926 in *PARP2*. However, these last results should be interpreted with caution given that the number of *BRCA2* carriers affected with ovarian cancer is four-fold lower than for *BRCA1* carriers and the statistical power was therefore more limited, increasing the possibility of false-positives. In the case of *PARP2*, imputed data showed a lower p-value of association (4×10^−4^) for another SNP, rs61995542, that had a slightly higher MAF than rs3093926 (0.074 vs. 0.067) (Figure S1). However, it must still be interpreted with caution due to small number of ovarian cancer cases in the *BRCA2* group.

It is worth noting that, four of the five genes for which strongest evidence of association was observed, are all DNA glycosylases participating in the initiation of BER by removing damaged or mismatched bases. Apart from the already mentioned *NEIL2* and *OGG1*, *TDG* initiates repair of G/T and G/U mismatches commonly associated with CpG islands, while *UNG* removes uracil in DNA resulting from deamination of cytosine or replicative incorporation of dUMP. We have not found strong associations with SNPs in genes involved in any other parts of the pathway, such as strand incision, trimming of ends, gap filling or ligation. It has been suggested that at least in the case of uracil repair, base removal is the major rate-limiting step of BER [33]. This is consistent with our findings, suggesting that SNPs causing impairment in the function of these specific DNA glycosylases could give rise to accumulation of single strand breaks and subsequently DNA double strand breaks that, in the HR defective context of *BRCA1/2* mutation carriers would increase breast and ovarian cancer risk.

The fact that the SNPs tested are located in genes participating in the same DNA repair pathway as PARP1, make them especially interesting, not only as risk modifiers but also because they could have an impact on patients' response to treatment with PARP inhibitors. *BRCA1/2* mutation carriers harboring a potential modifier SNP in DNA glycosylases could be even more sensitive to PARPi due to a constitutional slight impairment of the BER activity. This is a hypothesis that should be confirmed in further studies. The design of this study in two stages, the hypothesis-based approach adopted to select genes, and that it is based on the largest possible series of *BRCA1* and *BRCA2* carriers available nowadays, mean that the results obtained are quite solid However, the study still has some limitations such as the possible existence of residual confounding due to environmental risk factors for which we did not have information.

In summary, we have identified at least two SNPs, rs1466785 and rs2304277, in the DNA glycolylases *NEIL2* and *OGG1*, potentially associated with increased breast and ovarian cancer risks in *BRCA2* and *BRCA1* mutation carriers, respectively. Our results suggest that glycosylases involved in the first steps of the BER pathway may be cancer risk modifiers in *BRCA1/2* mutation carriers and should be more comprehensively studied. If confirmed, these findings could have implications not only for risk assessment, but also for treatment of *BRCA1/2* mutation carriers with PARP inhibitors.

## Materials and Methods

### Subjects

Eligible subjects were female carriers of deleterious mutations in *BRCA1* or *BRCA2* aged 18 years or older [6]. A total of 55 collaborating CIMBA studies contributed genotypes for the study. Numbers of samples included from each are provided in Table S2. A total of 1,787 mutation carriers (968 with mutations in *BRCA1* and 819 with mutations in *BRCA2*) from the CNIO, HCSC, ICO, Demokritos and MBCSG were genotyped in the first stage of the study. Stage II included 23,463 CIMBA samples (15,252 with mutations in *BRCA1* and 8,211 with mutations in *BRCA2*). All carriers participated in clinical and/or research studies at the host institution under IRB-approved protocols.

### Methods stage I

#### Selection and genotyping of SNPs

Eighteen genes (*UNG*, *SMUG1*, *MBD4*, *TDG*, *OGG1*, *MUTYH*, *NTHL1*, *MPG*, *NEIL1*, *NEIL2*, *APEX1*, *APEX2*, *LIG3*, *XRCC1*, *PNKP*, *POLB*, *PARP1 and PARP2*) involved in the BER pathway were selected, based on the information available at http://www.cgal.icnet.uk/DNA_Repair_Genes.html as at the 31^st^ December, 2009. Tag SNPs for the selected genes were defined using Haploview v.4.0 (http://www.broad.mit.edu/mpg/haploview) with an r^2^ threshold of 0.8 and a minimum minor allele frequency of 0.05. In addition, SNPs with potentially functional effects already described in the literature were selected. A final number of 144 SNPs was included in an oligonucleotide pool assay for genotyping using the Illumina Veracode technology (Illumina Inc., San Diego, CA). Three hundred nanograms of DNA from each sample were genotyped using the GoldenGate Genotyping Assay with Veracode technology according to the published Illumina protocol. Genotype clustering and calling were carried out using the GenomeStudio software. SNPs with a call rate <0.95 were excluded from further analysis. Duplicate samples and CEPH trios (Coriell Cell Repository, Camden, NJ) were genotyped across the plates. SNPs showing Mendelian allele-transmission errors or showing discordant genotypes across duplicates were excluded.

#### Statistical analysis

To test for departure from Hardy-Weinberg equilibrium (HWE), a single individual was randomly selected from each family and Pearson's X^2^ Test (1df) was applied to genotypes from this set of individuals. The association of the SNPs with breast cancer risk was assessed by estimating hazard ratios (HR) and their corresponding 95% confidence intervals (CI) using weighted multivariable Cox proportional hazards regression with robust estimates of variance [34]. For each mutation carrier, we modeled the time to diagnosis of breast cancer from birth, censoring at the first of the following events: bilateral prophylactic mastectomy, breast cancer diagnosis, ovarian cancer diagnosis, death or date last know to be alive. Subjects were considered affected if their age at censoring corresponded to their age at diagnosis of breast cancer and unaffected otherwise. Weights were assigned separately for carriers of mutations in *BRCA1* and *BRCA2*, by age and affection status, so that the weighted observed incidences in the sample agreed with established estimates for mutation carriers [1]; [34].

We considered log-additive and co-dominant genetic models and tested for departure from HR = 1 by applying a Wald test based on the log-HR estimate and its standard error. Additional independent variables included in all analyses were year of study, centre and country. All statistical analyses were carried out using Stata: Release 10 (StataCorp. 2007. Stata Statistical Software: Release 10.0. College Station, TX: Stata Corporation LP). Robust estimates of variance were calculated using the *cluster* subcommand, applied to an identifier variable unique to each family.

### Methods stage II

#### iCOGS SNP array

Stage II of the experiment was performed as part of the iCOGS genotyping experiment. The iCOGS custom array was designed in collaboration between the Breast Cancer Association Consortium (BCAC), the Ovarian Cancer Association Consortium (OCAC), the Prostate Cancer Association Group to Investigate Cancer Associated in the Genome (PRACTICAL) and CIMBA. The final design comprised 211,155 successfully manufactured SNPs of which approximately 17.5% had been proposed by CIMBA. A total of 43 SNPs were nominated for inclusion on iCOGS based on statistical evidence of association in stage I of the present study (p≤0.05). Of these, 36 were successfully manufactured and genotyped in CIMBA mutation carriers.

#### iCOGS genotyping and quality control

Genotyping was performed at Mayo Clinic and the McGill University and Génome Québec Innovation Centre (Montreal, Canada). Genotypes were called using Illumina's GenCall algorithm. Sample and quality control process have been described in detail elsewhere [32], [35]. After the quality control process a total of 23,463 carriers were genotyped for the 36 selected SNPs.

#### Statistical analysis

Both breast and ovarian cancer associations were evaluated in stage II. Censoring for breast cancer followed the same approach as in stage I. Censoring for ovarian cancer risk occurred at risk-reducing salpingo-oophorectomy or last follow-up.

The genotype-disease associations were evaluated within a survival analysis framework, by modelling the retrospective likelihood of the observed genotypes conditional on the disease phenotypes [9], [34], [36], [37]. The associations between genotype and breast or ovarian cancer risk were assessed using the 1 d.f. score test statistic based on this retrospective likelihood. To allow for the non-independence among related individuals, we accounted for the correlation between the genotypes by estimating the kinship coefficient for each pair of individuals using the available genomic data [34], [38], [39]. These analyses were performed in R using the GenABELlibraries and custom-written functions in FORTRAN and Python.

To estimate the magnitude of the associations (HRs), the effect of each SNP was modeled either as a per-allele HR (multiplicative model) or as genotype-specific HRs, and was estimated on the log-scale by maximizing the retrospective likelihood. The retrospective likelihood was fitted using the pedigree-analysis software MENDEL. The variances of the parameter estimates were obtained by robust variance estimation based on reported family membership. All analyses were stratified by country of residence and based on calendar-year and cohort-specific breast cancer incidence rates for mutation carriers. Countries with small number of mutation carriers were combined with neighbouring countries to ensure sufficiently large numbers within each stratum. USA and Canada were further stratified by reported Ashkenazi Jewish (AJ) ancestry.

#### Imputation

Genotypes were imputed separately for *BRCA1* and *BRCA2* mutation carriers using the v3 April 2012 release (Genomes Project et al., 2012) as reference panel. To improve computation efficiency we used a two-step procedure which involved pre-phasing in the first step and imputation of the phased data in the second. Pre-phasing was carried out using the SHAPEIT software [40]. The IMPUTE version 2 software was used for the subsequent imputation [41]. SNPs were excluded from the association analysis if their imputation accuracy was r2<0.3 or MAF<0.005 in any of the data sets. For the final analysis we only took in account those SNPs with an imputation accuracy r2>0.7, MAF>0.01 and being located in the region comprised within 15 kilo bases (kb) downstream and upstream the gene where the genotyped SNP showing an association was located (Table 1). Associations between imputed genotypes and breast cancer risk were evaluated using a version of the score test as described above but with the posterior genotype probabilities replacing the genotypes.

## Supporting Information

Figure S1 p-values of association (−log10 scale) with breast and ovarian cancer risk in *BRCA1* and *BRCA2* carriers for genotyped and imputed SNPs considering 15 kb upstream and downstream the genes in which SNPs described in Table 1 were located. rs numbers of SNPs from Table 1 are indicated at the top of each panel and in the graph with a purple arrow. For *PARP2* gene, the imputed SNP with the strongest association, rs61995542 is indicated with a red arrow. Colors represent the pariwise r^2^.
(PPT)

Table S1 Association with breast cancer for the 94 SNPs selected for analysis in stage I.
(XLS)

Table S2 number of *BRCA1* and *BRCA2* carriers by study.
(XLS)
