# Supplementary material for: DNA Glycosylases Involved in Base Excision Repair May Be Associated with Cancer Risk in BRCA1 and BRCA2 Mutation Carriers
Source: PLoS Genet. 2014 Apr 3;10(4):e1004256. doi: 10.1371/journal.pgen.1004256 (PMC3974638; doi:10.1371/journal.pgen.1004256)
Supplement: Figure S1 — p-values of association (−log10 scale) with breast and ovarian cancer risk in BRCA1 and BRCA2 carriers for genotyped and imputed SNPs considering 15 kb upstream and downstream the genes in which SNPs described in Table 1 were located. rs numbers of SNPs from Table 1 are indicated at the top of each panel and in the graph with a purple arrow. For PARP2 gene, the imputed SNP with the strongest association, rs61995542 is indicated with a red arrow. Colors represent the pariwise r2. (PPT) [file pgen.1004256.s001.ppt]

## Slide 1
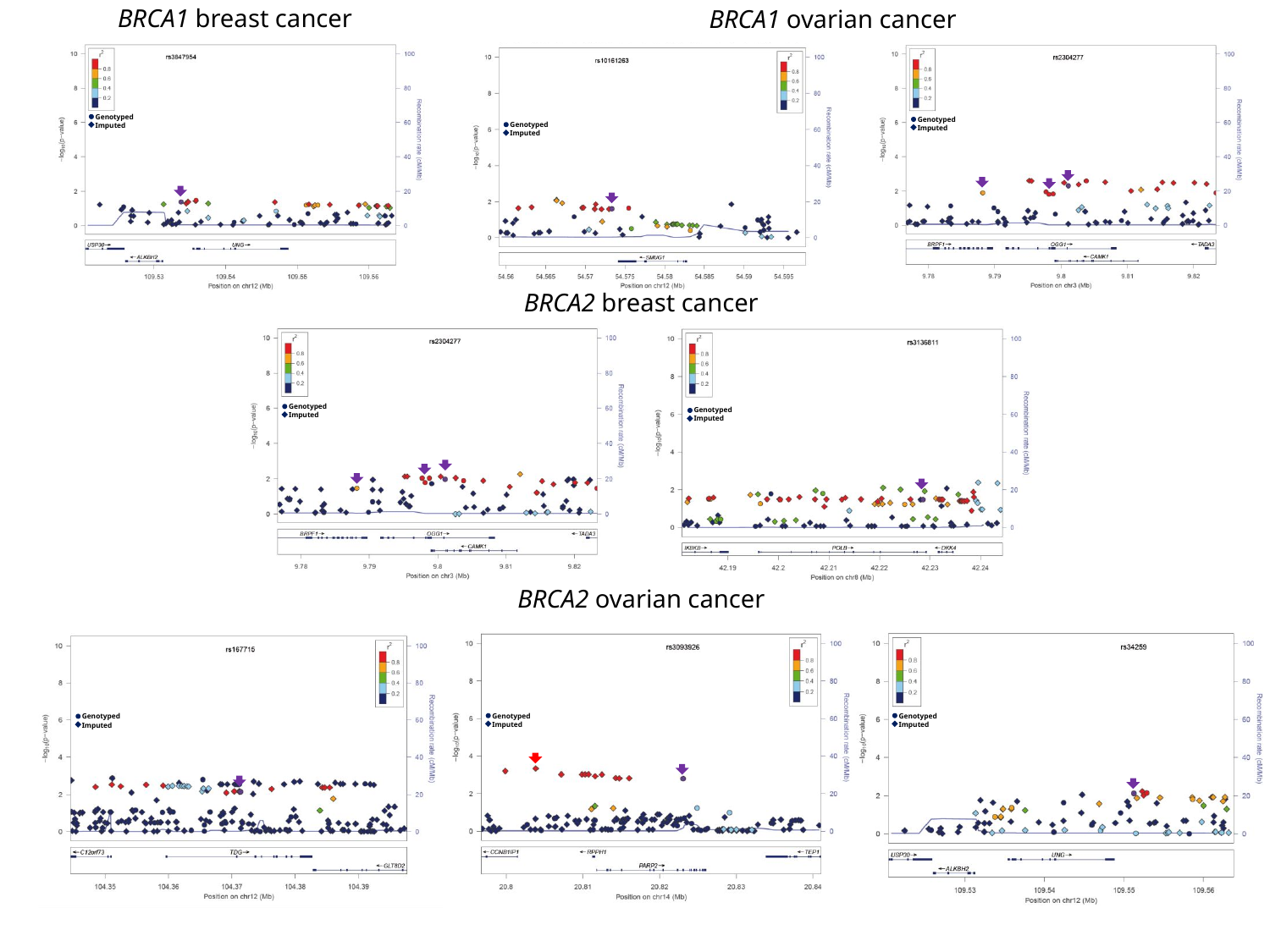

BRCA1 breast cancer
BRCA1 ovarian cancer
Genotyped
Imputed
Genotyped
Imputed
Genotyped
Imputed
BRCA2 breast cancer
Genotyped
Imputed
Genotyped
Imputed
BRCA2 ovarian cancer
Genotyped
Imputed
Genotyped
Imputed
Genotyped
Imputed
